# A Generative Model for Measuring Latent Timing Structure in Motor Sequences

**DOI:** 10.1371/journal.pone.0037616

**Published:** 2012-07-16

**Authors:** Christopher M. Glaze, Todd W. Troyer

**Affiliations:** 1 Department of Biology, University of Pennsylvania, Philadelphia, Pennsylvania, United States of America; 2 Department of Biology, University of Texas at San Antonio, San Antonio, Texas, United States of America; Claremont Colleges, United States of America

## Abstract

Motor variability often reflects a mixture of different neural and peripheral sources operating over a range of timescales. We present a statistical model of sequence timing that can be used to measure three distinct components of timing variability: global tempo changes that are spread across the sequence, such as might stem from neuromodulatory sources with widespread influence; fast, uncorrelated timing noise, stemming from noisy components within the neural system; and timing jitter that does not alter the timing of subsequent elements, such as might be caused by variation in the motor periphery or by measurement error. In addition to quantifying the variability contributed by each of these latent factors in the data, the approach assigns maximum likelihood estimates of each factor on a trial-to-trial basis. We applied the model to adult zebra finch song, a temporally complex behavior with rich structure on multiple timescales. We find that individual song vocalizations (syllables) contain roughly equal amounts of variability in each of the three components while overall song length is dominated by global tempo changes. Across our sample of syllables, both global and independent variability scale with average length while timing jitter does not, a pattern consistent with the Wing and Kristofferson (1973) model of sequence timing. We also find significant day-to-day drift in all three timing sources, but a circadian pattern in tempo only. In tests using artificially generated data, the model successfully separates out the different components with small error. The approach provides a general framework for extracting distinct sources of timing variability within action sequences, and can be applied to neural and behavioral data from a wide array of systems.

## Introduction

There has been an increasing focus in systems neuroscience on the role of trial-to-trial variability in the neural code for movement [1]–[3]. Motor variability has been of particular interest in songbird research, including studies on zebra finch song: males exert active control over song variability, singing more stereotyped song when courting a female than when singing alone [4]–[7]. Further experiments have shown that this control is largely mediated by the anterior forebrain pathway, a cortical-basal ganglia circuit for song [5]–[8].

Interestingly, single unit recordings in singing birds reveal that bursting in individual neurons is locked to song output with millisecond precision [9]–[11], with trial-to-trial variability in inter-burst intervals being strongly correlated with variability in the timing of corresponding song features [10]. Several studies have exploited this relationship and used precise measurements of song timing to make inferences about the nature and timescale of the underlying motor code [12]–[15].

However, studies aimed at linking brain and behavior face the challenge that variability is likely to be driven by multiple sources with different timescales and mechanistic explanations. For example, a natural way to measure timing variability would be to measure the standard deviation of individual song elements known as “syllables.” While some of that timing deviation is likely due to fast neural and synaptic noise within the central circuits that generate song timing, variability in syllable length is also likely to be affected by slower modulations that alter song tempo. Finally, alterations in timing introduced downstream from the song pattern generator, either in motor periphery or by measurement errors in syllable duration, will contribute to variability in measured syllable duration as well. To understand the mechanisms of song production one would like to separate these distinct components of timing variability.

We have developed a statistical model to address this problem, allowing researchers to distinguish three kinds of timing variability in repeated action sequences such as zebra finch song: global timing changes spread across the sequence (“tempo”), fast, uncorrelated timing noise in the length of each element of the sequence (“independent variability”), and timing “jitter” at the onsets and offsets of sequence elements that might be caused by variation in the motor periphery or by measurement error. This “timing variability model” can be seen as an extension of the Wing-Kristofferson model [16], which separates central and peripheral components of variability in human interval timing during a finger-tapping task. Our approach uses a novel variation on factor analysis, a technique for detecting and measuring latent variables in high-dimensional data sets [17]–[19]. We fit the model to data using an expectation-maximization (EM) algorithm, which allowed us to estimate the contribution of each of the three latent factors to the variability of each element of the sequence.

We applied the model to the songs of 11 adult male zebra finches and found that for most birds the model provides very good fits to trial-to-trial variability in the durations of individual song vocalizations (syllables) and the gaps of silence between them. For individual syllables and gaps, a roughly equal amount of variability is contributed by each of the three components of timing variability. For 5 birds, the model appears to be optimal when including an additional latent variable in which syllable and gap durations are anti-correlated. We also performed Monte Carlo simulations to investigate how accurately the model was able to fit artificial data sets where the underlying timing parameters and latent variables were known.

## Results

This paper develops a statistical model to estimate latent factors governing trial-to-trial timing variation in repeated action sequences. We will present our results in three sections. First, we describe our model which we call the “timing variability model.” We then fit the model to timing data from zebra finch songs, and present several results regarding the nature of timing variability in this species. Finally, we describe Monte Carlo simulations designed to quantify the reliability of the model fits.

### Timing Variability Model

**Figure 1:**
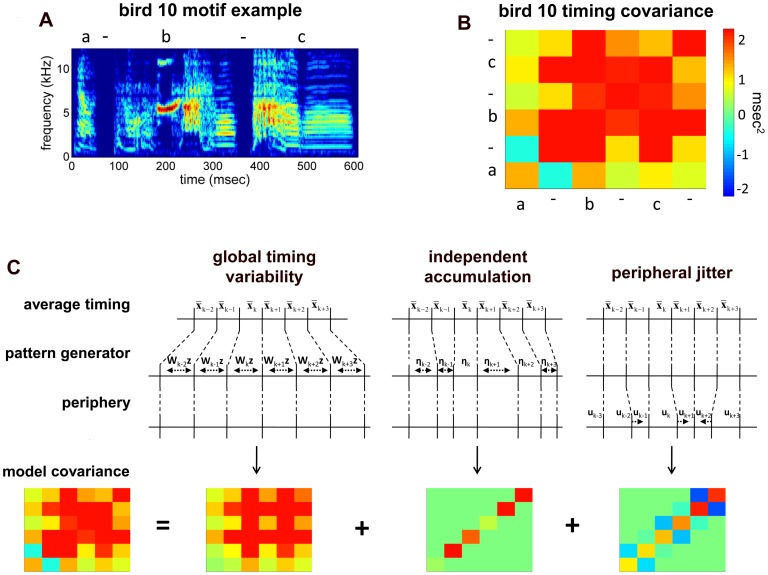
Timing variability and its decomposition. A, Spectrogram of a single song motif from one bird, with syllables indicated above by letters and silent gaps by “-”. B, timing covariance matrix for the same bird, in which the color of each square indicates the pairwise covariance between the durations of two intervals denoted along the rows and columns. C, Schematic of the different noise sources captured in the model. Below each noise source is a covariance matrix generated by the model of that source.

The timing variability model can be applied to data consisting of repeated action sequences that can be divided into consecutive time intervals, as might be observed during the production of a stereotyped behavior over multiple trials. Although the model may be applied to any action sequence, here we apply the model to quantify timing variability in the songs of male zebra finches. Each adult produces a song that is highly invariant but unique to that male (Fig. 1A). The song of each male consists of a stereotyped string of 3–7 distinct vocalizations we term “syllables” that vary in duration from approximately 50 to 200 msec; syllables are separated by gaps of silence that are roughly half the duration of syllables. We will use the term “motif” to denote a male’s stereotyped string of syllables, while a song “bout” consists of a series of motifs produced back-to-back. We restricted the analysis to the first motif of each bout; however, to examine the gap of silence between the 1st and 2nd motifs, we included the first syllable of the second motif in the sequences analyzed. We will use the term “time interval” to denote the durations of both syllables and silent gaps, so if a sequence consists of *L* syllables, it also contains 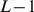 gaps and thus 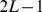 time intervals. The duration of each syllable and silent gap is also fairly stereotyped, varying across motif renditions by about 1.5–3.5 msec standard deviation in duration, or ∼2–5% coefficient of variation (CV) [15]. This temporal stereotypy gives each male’s song a very characteristic rhythm [15], [20].

#### Variance decomposition

To start we describe the model from the perspective of variance decomposition, generally following the standard approach known as factor analysis [17], [19], [21], [22]. As outlined in the introduction, we would like to write the variance in interval length as a sum of three component variances: variance in tempo spread across the song, independent variance that is specific to each timing interval within the song, and timing jitter in the exact placement of the onset and offset of each song syllable.

For the first factor, we presuppose a non-observable, latent factor 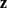 that influences song tempo, with 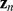 representing the deviation in tempo from its mean value on each trial 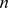. If the change in tempo is spread uniformly over the song, the deviation in the length of interval *k* will be proportional to the mean length of that interval, 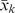; that is, the deviation will equal 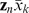. However, the timing variability model is not restricted to uniform changes in tempo. Rather, deviations in global timing are modeled as 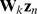 where the values 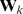 are free parameters representing the relative sensitivity of each interval to variations in global timing. For that reason, we refer to 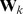 as the “global weight” for interval *k*. Under these assumptions, variations in global timing will contribute a variance component to interval *k* equal to 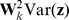 where 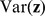 represents the variance of the latent factor **z**. Importantly, global timing will also result in a covariance between interval *j* and interval *k* equal to 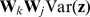. By fitting this pattern of covariances to the off-diagonal elements of the data covariance matrix, the model forms an estimate of the global sensitivity of each interval to global timing (Fig. 1C, left).

A similar approach can be used to estimate the variance contributed by timing jitter. For this component, we presume that the length of each interval is set centrally, but the measured timing of the transition between consecutive intervals is subject to variation downstream of central pattern generator for song, either in the motor periphery or during the measurement process. We let 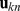 denote the jitter in timing between interval *k* and 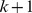 on trial 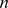. As noted by Wing and Kristofferson [16] in the context of human finger-tapping experiments, such timing jitter introduces a negative covariance between adjacent intervals in addition to contributing to the variance in interval duration (Fig. 1C, right). As before, off-diagonal values in the data covariance matrix can be used to estimate the magnitude of timing variance at each boundary in the sequence. We denote the variance at the 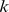
th boundary by 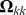. The variance contributed by jitter the timing of interval 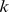 will be 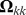
+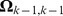.

Finally, we assume that each interval *k* is subject to its own degree of independent variation 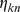 on trial *n*. This independent variance can be estimated by calculating the total interval variance and subtracting the variance contributed by global and jitter factors. We denote the independent variance for interval *k* by 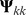.

Variance decomposition in the model is shown in figure 1. Panel B shows the data covariance matrix from a single bird. Panel C shows the three matrices of covariance contributed by the global, independent and jitter terms in the model to the right and how these sum to equal the model covariance matrix at the left.

The difference between the timing variability model and traditional factor analysis stems from the explicit incorporation of the negative covariances between adjacent intervals caused by timing jitter at syllable onsets and offsets. It should be noted that since the model can only estimate jitter by recognizing negative covariation between adjacent intervals, estimates of timing variability in the first and last intervals of the sequence will be inherently less accurate. As a result, we discarded the first and last intervals in the sequence from all reported analyses.

#### Generative model and maximum likelihood estimation

We used a maximum likelihood approach to fitting our model to the data [17], [22], [23]. More specifically, we view the timing variability model as a probabilistic model capable of generating artificial data consisting of 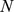 repeated sequences, each containing the duration of 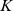 consecutive time intervals. To generate such data, we assume that the parameters of the model are fixed and for each artificial sequence 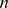 we generate random values for the unobserved, latent variables: a single global timing variable, 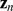, independent variations for each of 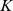 intervals, 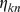, and jitter variations at each of the 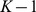 boundaries between intervals, 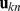. These are combined to produce 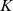 observed interval durations
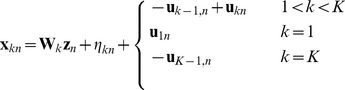
(1)For convenience we can rewrite this equation in vector notation as:
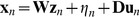
(2)where 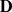 is the 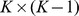 differencing matrix with ones along the diagonal (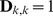 since boundary 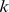 causes deviations in the offset of interval 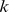), negative ones along the subdiagonal (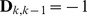 since boundary 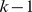 causes deviations in the onset of interval 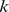), and 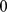 elsewhere.

To allow theoretical derivations of maximum likelihood fits, we assume that the latent factors are independent and Gaussian distributed. The free parameters of the model are then the global weights in 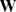 (collectively referred to as the global parameters) and the variances of 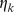 and 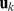. We let 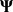 and 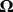 be the covariance matrices of the random vectors 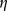 and 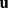 respectively; these matrices are diagonal since the latent variables in 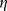 and 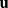 are assumed to be independent. The variance of the global parameter 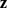 is fixed at a value of one, with the magnitude of the global weights 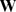 determining the magnitude of the observed variances. Letting **S** be the covariance matrix of interval durations, and using the basic result that 
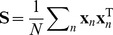
, equation 2 implies that in the limit of large *N*, the timing variability model will generate *K* dimensional Gaussian distributed data with covariance matrix
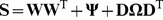
(3)In particular, for any given sequence of interval durations, we can calculate the probability that the timing variability model would generate that particular sequence.

To fit the model to observed data, we find parameters 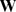, 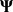 and 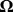 that maximize the likelihood that the model would generate the observed collection of sequence durations. To accomplish this, we use an Expectation-Maximization (EM) algorithm that was modified from the standard EM algorithm used to generate maximum likelihood fits for standard factor analysis [17], [23]. (See Methods for details.) A consequence of this maximum likelihood, generative approach is that the EM algorithm naturally yields estimates of the latent variables 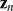, 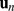 and 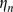 on a trial-to-trial basis. As we will demonstrate below, this allows for an analysis of the relationship between timing factors and other variables, such as time of day and behavioral state, that vary across samples but are not explicitly included in the model. Furthermore, the model can be used to generate artificial data sets from a model with known parameters. These artificial data sets can then be fit using the same class of model. This exercise allows one to estimate the uncertainty in parameter estimates that are due solely to statistical variation in finite data sets (see ‘Monte Carlo experiments’ below).

### Timing Variability in Zebra Finch Song

We applied the timing variability model to song samples from 11 adult zebra finch males reported in a previous study [15]; one bird from the previous study was omitted because the sample was not large enough to yield reliable fits. For each male we collected a sample of 215–885 song bouts recorded while the bird was alone, housed with another adult male, or serving as a song tutor to a juvenile. Data from individual birds often included recordings from multiple recording sessions, spanning periods that ranged from 1–6 months. We restricted the analysis to the first motif of each bout; however, to examine the gap of silence between the 1st and 2nd motifs, we included the first syllable of the second motif in the sequences analyzed. We will refer to gaps between motifs as “inter-motif gaps” and distinguish them from “within-motif gaps”. After fitting the model to the data, we then excluded the first and last syllable of each sequence since the parameter estimates for these intervals are corrupted by unknown jitter at the beginning and end of the sequence (see above). The resulting data set included parameter estimates for 48 syllables, 48 within-motif gaps and 11 between-motif gaps, for a total of 107 time intervals. Throughout we will report parameter distributions with median
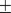
median absolute deviation (MAD).

#### Model fit

**Figure 2:**
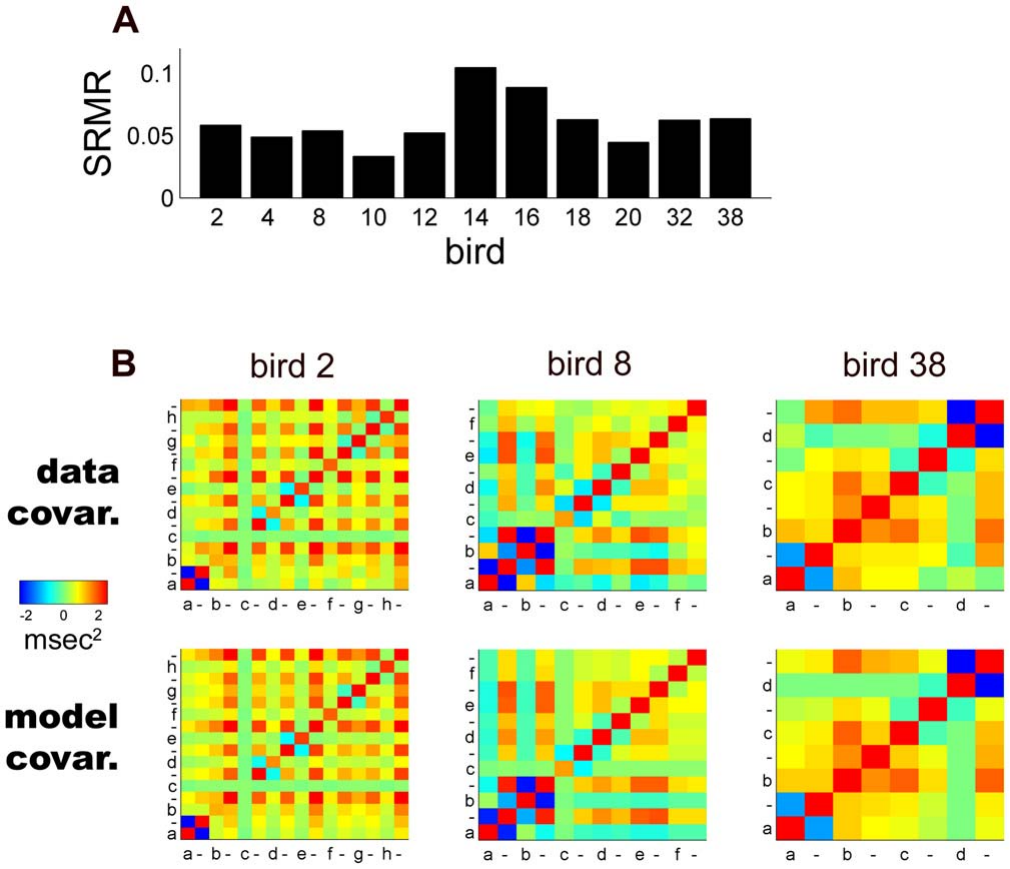
Goodness of fit between the timing model and data from 11 zebra finch birds. A, SRMR by bird (see Results). B, Color plots of the timing covariance matrices from the data and model for 3 representative birds.

We assessed the overall fit of the model to the data using the standardized root mean-squared residual (SRMR) [24]. This measure is based on the average difference between the pairwise interval length correlations in the data and those predicted by the model covariance matrix (see Methods for formula). As a rule of thumb in factor analysis, a SRMR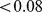 is considered an adequate fit while SRMR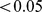 is considered an excellent fit [24]. Across birds, SRMR was a median 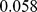, range of 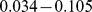. Nine of eleven birds yielded an SRMR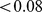 while 3 yielded SRMR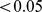 (Fig. 2A). Thus, the model fit a large majority of birdsongs in our sample reasonably well. The particularly large SRMR values appeared to be due to timing factors not included in the original model but analyzed below.

#### Parameter distributions and the contribution of tempo

Across all time intervals, median global variability was 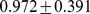 msec, median independent variability was 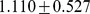 msec, and median boundary jitter was 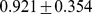 msec. Averaging across intervals produced by a given bird, respective medians ranged from 0.550–1.456 msec (global), 0.560–2.348 msec (independent), and 0.638–1.723 msec (jitter). Thus, each of the three timing factors made similar contributions to the variation in duration of individual song elements. In general, timing variability was relatively small in these songs. It may also be noted that, when respective variances are summed to estimate total duration variability in song elements (assuming an interval receives jitter variance from two sources), the numbers agree with previously reported data: 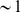 msec^2^ variance in each component yields 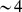 msec^2^ interval variance total (so 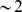 msec st. dev.), on par with what we have previously reported [15].

Based on a previous analysis showing generally positive correlations among the durations of time intervals, we expected global timing variations in which most time intervals shortened or lengthened together [15]. Of the 107 song intervals analyzed, only 4 had global weights <0, with two of the negative weights coming from a single bird (out of 11 time intervals for that bird). Since nearly all global weights were positive, global timing variations indeed were dominated by changes in which time intervals shortened or lengthened together. Therefore, the global factor represents changes in a shared tempo across the song and we will use the terms “global variability” and “tempo variability” interchangeably.

Although global weights were on the same order of magnitude as the independent variability for individual intervals, the variance in total motif length was dominated by tempo changes. Because global tempo is shared by all song elements, the motif length variance linked with the global variable (equal to 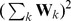) was roughly 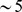 times as much as the length variance linked with the independent variable (equal to 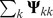). More specifically, tempo explained 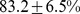 of the total variance in motif duration explained by the model (range 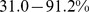 by bird). Only one bird (bird 4) had more motif length variance linked with the independent variable than with the global; interestingly, this appeared to be entirely due to independent variance within a silent gap corresponding to a variable sequence point at which the bird occasionally transitioned to a different syllable.

Overall, the motif length variance in the model (equal to the sum of global and independent variance) deviated from measured motif length variance by only 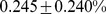 across birds (maximum of 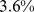).

#### Length scaling

A wide range of studies, from human finger-tapping experiments to previous work in birdsong, have found that variability in the duration of a learned motor gesture tends to scale with the average duration of the gesture [13], [15], [16], [25]–[28]. This is to be expected from any process that consists of a causal sequence of activity with noise at each time point: the longer the process, the more variability accumulates. We hypothesized that variability would scale with average duration for the tempo and independent timing parameters, as these parameters are expected to alter timing throughout the duration of a given interval. In contrast, we did not expect any relationship between average duration and the variability linked with boundary jitter. To quantify the boundary jitter linked with interval *k*, we calculate the square root of the sum of jitter variances at the two ends: 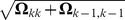.

**Figure 3:**
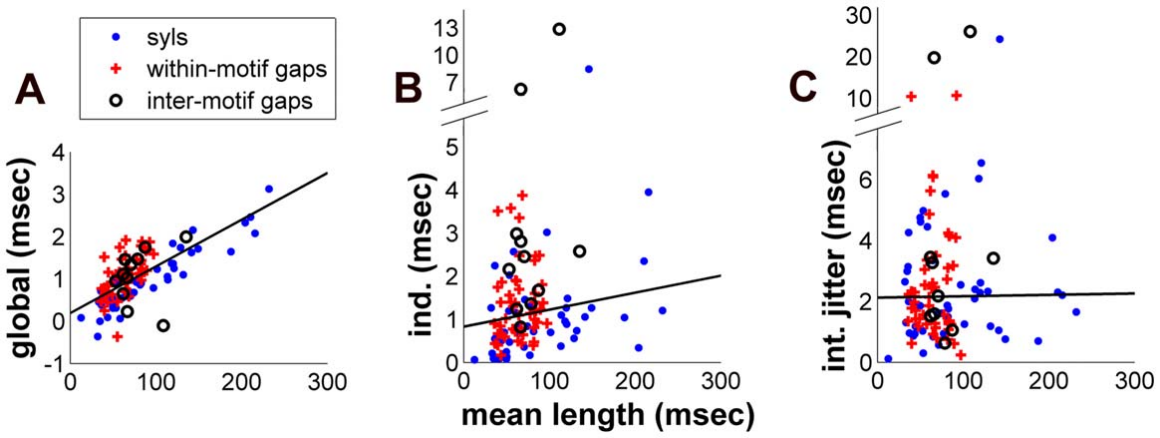
Scaling of timing parameters with average length. A, Scatter plot of global tempo variability by average interval duration across all syllables and silent gaps. Silent gaps within motifs and between motifs have been separated out. B, C, Analogous plots for the independent and jitter parameters respectively. In each plot solid lines come from a regression of timing parameters on average interval duration, calculated across birds. Overall, plots show that global and independent variability scale with average duration, while jitter does not. The plots also show systematic differences among interval types with respect to how much each parameter scales with average duration.

Indeed, the data confirmed that only tempo-based and independent variability scale with average interval duration (Fig. 3): the correlation between global variability and average interval duration was 0.701 (Spearman, 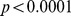), between independent variability and duration, 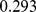 (Spearman, 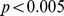). In contrast, average duration showed no significant correlation with jitter-based variability (Spearman’s 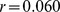, 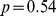). Although the relationship between independent variability and average duration was significant, it was far weaker than what we found with tempo-based variability. There are a number of reasons why this might be, including greater error in independent parameter estimates as well as real factors in the neural system that may contribute noise in a way that is not dependent on process duration.

#### Model parameters distinguish syllables from silent gaps

Previous research has shown that silent gaps stretch and compress with song tempo more than syllables after dividing out average duration, *i.e.* they are more “elastic” to trial-to-trial tempo variability [13], [15] (although see [12]). We examined whether the global parameters from our model indicated the same difference between syllables and gaps. Here, we computed tempo elasticity for interval 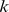 as 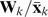, where 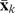 is the mean duration of the 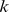
th time interval. This measure may be viewed as a partial CV with respect to tempo so we will occasionally refer to it as “global CV”. For example, a global CV of 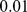 in an interval would indicate that the standard deviation of global timing expressed in the interval is 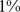 of its average duration.

Indeed, the model verified that syllables were less elastic to tempo than gaps (Fig. 3A), with most gaps falling above the regression line of global variability on mean duration and most syllables falling below. Syllable tempo elasticity was 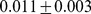, while gap tempo elasticity was about 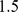 times greater on average, 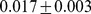 (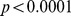, Wilcoxon rank-sum test).

Although previous research has focused on elasticity with respect to shared, tempo-based variability, our timing variability model also allowed us to investigate the pattern of sensitivity to independent variability as well. (We did not analyze jitter differences because syllables and gaps share the same set of interval boundaries and there is no relationship with average duration to begin with.) Because the independent parameters scaled with length we derived an analogous quantity that may be viewed as a partial CV with respect to independent variability, 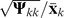. Interestingly, here we found that as with tempo CV, the independent CV was smaller for syllables than for gaps (Fig. 3B): independent CV was 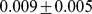 among syllables, and about twice as great on average among gaps, 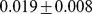 (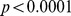, Wilcoxon rank-sum test).

#### Inter-motif gaps express more independent variability

In a hierarchically arranged motor code the onsets of entire motifs may be triggered by mechanisms that are different from what triggers the onsets of individual syllables. There is electrophysiological evidence for this segregation [29], while previous analysis has suggested increased timing variability at motif boundaries [15]. Therefore, we examined whether the variability in duration of silent gaps between motifs differed from the gaps between syllables within a motif (Fig. 3A–B). Indeed, the 11 inter-motif gaps we investigated had a significantly greater independent CV, 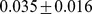, vs. 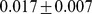 among within-motif gaps (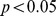, Wilcoxon rank-sum test). Interestingly, we did not find the same difference with respect to global CV: inter-motif gaps had a median 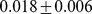 vs. 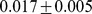 among within-motif gaps (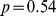, Wilcoxon rank-sum test).

Thus, the model revealed timing properties that systematically vary by interval type, with gaps expressing proportionally greater tempo changes and independent variability, and inter-motif gaps expressing an especially large amount of independent variability.

### Circadian Influence and day-to-day Drift in Latent Timing Variables

The analyses above examined the relative magnitudes of three components of timing variability and their distribution across song elements. One advantage of using the EM algorithm is that it naturally provides maximum likelihood estimates of the latent variables on a trial-by-trial basis. This allows investigations in which these unobservable, latent factors are correlated with other biologically relevant variables. For example, changes in song timing can be tracked over periods ranging from hours to days and even months.

Here, we investigated whether any of the latent timing variables underwent consistent patterns of modulation over the course of the day [15] or drifted over the 1–6 months of song recording. Specifically, for each latent variable and each bird we used a Bonferroni corrected two-factor ANOVA to test for significantly different averages by both hour of the day and day of song production. If we found significant differences, we then examined the pattern of changes related to a given factor by calculating a mean and standard error of the latent variable; we adjusted these estimates for unequal sample numbers across factor combinations using the “multcompare” function Matlab.

**Figure 4:**
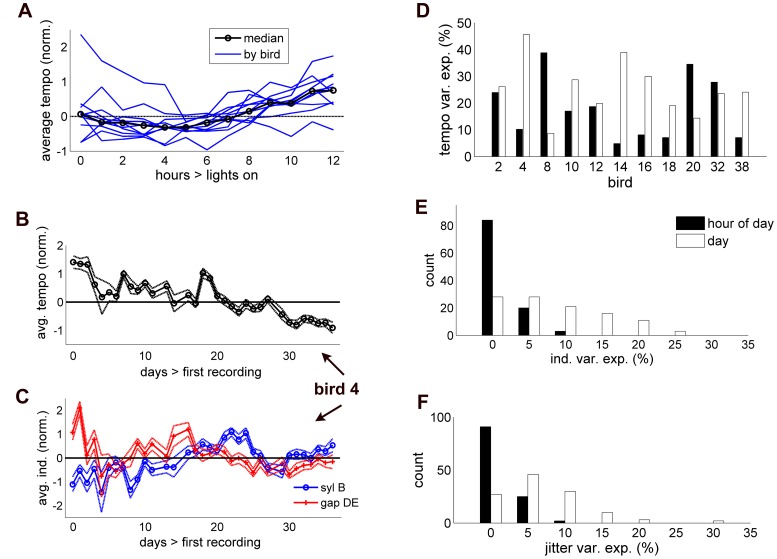
Circadian rhythm and day-to-day drift in the latent timing variables. A, Average tempo by hour of the day (defined from lights on) by bird (blue lines) and averaged across birds (black). B, example of day-to-day drift in tempo for one bird over a 1-month period. C, example of drift in independent variability for two different song segments in the same bird. All data in plots A-C are group means from a two-factor ANOVA that have been adjusted for unequal samples of factor combinations (see Results), while dotted lines in B and C indicate adjusted means ± standard error. D, The estimated amount of variance in tempo explained by both hour of the day (black) and day (white) across all 11 birds. E and F, histograms of the amount of variance in the independent and jitter variables respectively, explained by both hour of the day and day (same color code). Generally the data show significant circadian variation in tempo only, and day-to-day drift in all three latent timing variables.

All 11 birds showed significant tempo changes that were affected by both time of day and day of singing (Fig. 4A,D), indicating both a circadian variation and slow drift in song tempo (Bonferroni-corrected 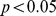). Across birds, the amount of variance explained by hour of the day was a median 17.0±9.9% (range 4.9–38.8% by bird), by daily drift, 24.1±5.1% (8.7–45.6%). Consistent with our previous study [15], the pattern of circadian variation in tempo was similar across birds (Fig. 4A), with songs speeding up until mid-morning and slowing down over the afternoon.

The model also allowed us to test for circadian changes and day-to-day drift in the independent and jitter latent variables 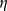 and 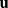 (Fig. 4B,C,E,F). In contrast to tempo, neither showed consistent changes by hour of the day, with only 5 of 107 independent components and 3 of 118 jitter components showing significant change by that factor (Bonferroni-corrected 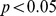). Interestingly, many intervals did show significant drift in those variables, with 54/107 independent and 50/118 jitter components reaching significance (Bonferroni-corrected 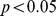). Of the intervals with significant drift, that factor explained 13.0±4.1% of the independent variation (range 3.1–23.4%) and 9.7±2.4% of the boundary jitter (range 3.5–30.5%).

Thus, the data indicated day-to-day drift in all three types of latent timing variables, and a significant circadian pattern in tempo only.

### Multiple Global Timing Factors

Our original construction of the model incorporated a single global factor, motivated by an a priori expectation of variation in song tempo which was confirmed by the data. However, the factor analysis framework also allowed us to search for additional global factors whose influence is spread across the song sequence. The existence of such additional factors was suggested by a qualitative examination of the data from several birds as well as correspondingly high SRMR values for those model fits. The EM algorithm easily accommodated the inclusion of multiple global factors simply by increasing the number of dimensions in 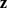 and 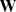 and fitting parameters as before (see Methods).

**Figure 5:**
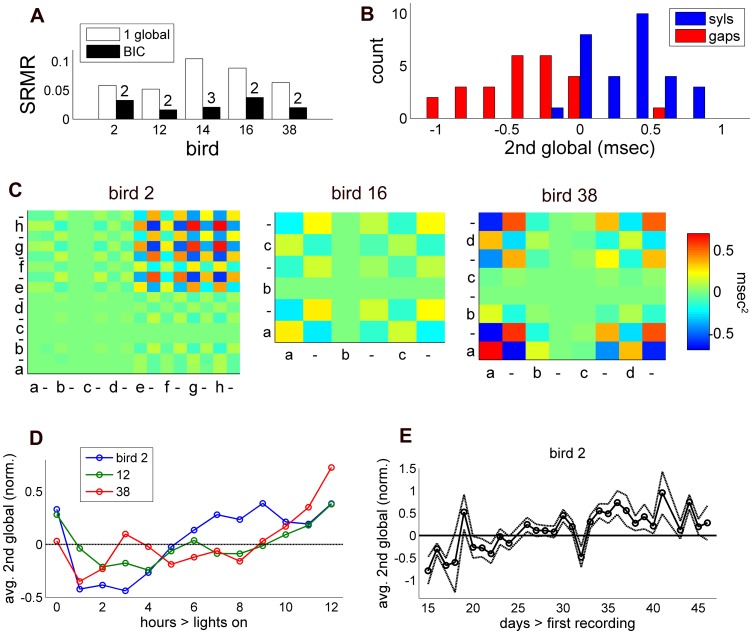
The influence of additional global factors on model fit and song timing. A, Goodness of model fit with additional global factors included (black) vs. 1 global factor (white) for the 5 birds for which BIC was lowest for 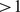 factor. Numbers above black bars indicate the number of factors associated with the lowest BIC for that bird. B, Distribution of global parameters for the 2nd factor (sign-normalized, see Methods), separated by whether the song segment was a syllable or silent gap. Across birds syllable and gap parameters tended to be of opposite sign. C, Timing covariance matrices generated for the 2nd global factor in 3 representative birds. D, Average changes in the 2nd global variable in the 3 birds that showed significant circadian variation for that factor. The circadian pattern showed that syllables tended to elongate at the expense of gaps over the afternoon. E, Example of day-to-day drift in the 2nd global variable for one bird over a 1-month period. As in figure 4, hour-of-day and daily averages in D and E have been adjusted for unequal sampling of factor combinations while dotted lines in E represent adjusted means ± standard error.

By adding parameters to our model, we are assured of improving the fit to the data. To avoid over fitting, we ran models with 1–4 global dimensions, and used the minimum Bayesian Information Criterion (BIC; see Methods) to determine the optimal tradeoff between better fits to the data and model complexity. Across our sample, 6 of 11 birds had a BIC lowest for just 1 global factor. Of the remaining 5 birds, 4 produced models that had a lowest BIC for 2 global factors while the remaining bird had 3 global factors. As expected, additional global factors improved the fit to the data: of the 5 birds with 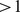 global factor, median SRMR dropped from 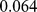 to 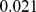 (Fig. 5A); across all birds, median SRMR after using the BIC was 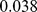, range 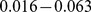.

As in traditional multi-dimensional factor analysis, the model fit can only determine the subspace spanned by the matrix 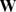. Within this subspace there are an infinite number of variations of 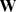 that will give the same pattern of covariances in 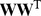. Based on our previous analysis with one global factor, we sought a solution that separated out tempo-based variability from any additional timing factors. In particular, we used a transformation of 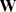 in which global factors affecting total sequence length would be confined to the 1st dimension, with additional timing factors chosen orthogonal to this tempo dimension (see Methods). In the one bird with 3 global factors, we chose as the 2nd factor the one which explained the most interval variance after factoring out the tempo dimension. We then analyzed the distributions of parameters from the 2nd global factor across the 5 birds with 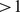 global dimension.

The second factor tended to have weights of the opposite sign across syllables and silent gaps (Fig. 5B,C). Since factors are ambiguous with respect to overall sign, the second global factor was chosen so that the sum of the syllable weights was positive. With that convention, 26 of 30 syllable weights were 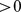 and 23 of 25 gap weights were 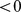. Median syllable weight was 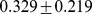 msec, vs. 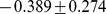 msec across gaps (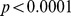, Wilcoxon rank-sum test), and the difference was consistent across all 5 birds. Interestingly, this pattern corroborates a previous finding of significant length correlations in syllable-syllable and gap-gap pairs after subtracting out the influence of tempo [15]. Unlike the tempo-based weights, we found no relationship between magnitude of the weight for the second component and the mean length of the interval (Spearman’s 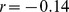, 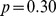). Thus the second global factor showed properties that were similar to jitter: it yielded a tradeoff between syllable and gap lengths and did not scale with average interval duration.

In all 5 birds, the second global variable showed a significant daily drift, and there was a significant circadian component in 3 of 5 birds (Bonferroni-corrected two-way ANOVA, 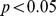; Fig. 5D,E). Hour of day explained 25.0–50.4% (median 28.5%) of the variance in the 3 birds for which the circadian component was significant, while daily drift explained 39.1–69.0% (median 45.1%) across the 5. The trend in the circadian component was for syllables to get longer and the expense of gap duration over the afternoon. Overall, these longer timescales explained a remarkably large amount of variance in the component.

Finally, we examined whether the inclusion of additional global factors significantly changed any parameter estimates from the original model with only 1 global dimension. Among the 5 birds with 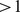 global factor included from the BIC analysis, tempo-based weights changed only slightly, with the median decrease of 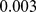 msec failing to reach significance (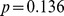, Wilcoxon signed-rank test). The median absolute magnitude of the change was 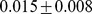 msec, much smaller than the median weight of 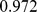 msec. Independent parameters changed more, with median absolute change of 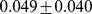 msec. However, as with the tempo weights, the direction of change was variable so that the median change of 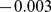 msec over all intervals was not significant (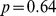, Wilcoxon signed-rank test). Jitter parameters also showed a larger absolute change of 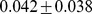 msec. But unlike independent parameters, jitter parameters tended to get smaller, with 34 of 50 boundaries showing decreased jitter, resulting in a small but statistically significant median decrease of 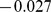 msec (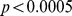, Wilcoxon signed-rank test). This suggests that a small amount of the estimated boundary jitter in the single-global factor model may be explained by the song-wide anti-correlation between syllables in gaps that was uncovered in the 2nd global factor.

### Monte Carlo Experiments

One of the difficulties in using latent variable models is that the model fit cannot be directly validated by comparison with observed data. As an alternative, we investigated how accurately the model was able to fit artificial data sets where the underlying timing parameters and latent variables were known. Specifically, for each bird we selected parameter values that were fit to the experimental data. Then using the model in generative mode, we created an artificial data set with the same number of samples by randomly generating latent variables from a unit normal distribution, scaling the independent and jitter variables by the corresponding parameters, and combining these into observed interval lengths according to equation (1). Focusing exclusively on this artificial data set, we reapplied our algorithm to find the parameters that provided the best fit to the artificially generated data. In order to derive statistics of how well the model fit the artificial data, we generated 200 distinct artificial data sets for each bird.

**Figure 6:**
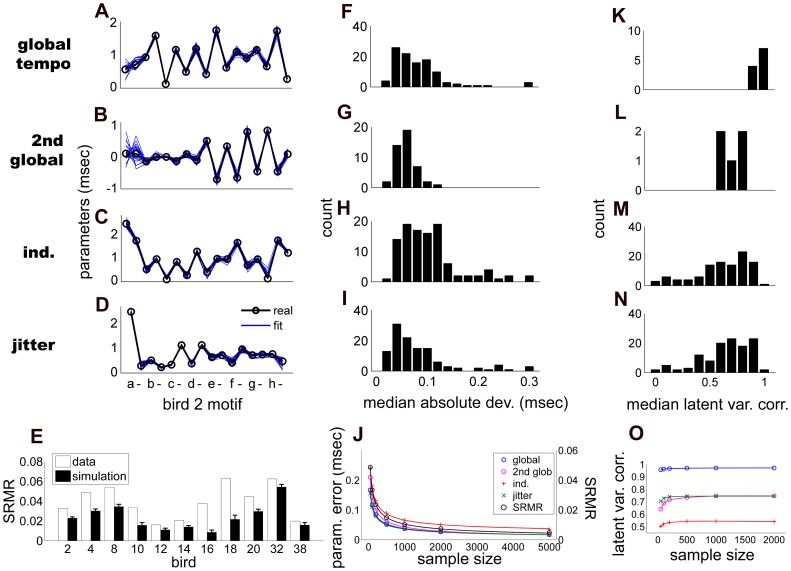
Results of Monte Carlo simulations based on real parameter distributions in the song data. For plots A-D, F-I and K-N, parameter type is designated by labels on the far left. A-D, Real parameter values (black lines), along with parameter estimates from 20 randomly selected simulations (blue) for one bird. E, Median SRMR across simulations for each bird (black bars, errorbars indicate median absolute deviation) vs. SRMR values from the real data (white bars). F-I, Median absolute deviation in parameter estimates from real parameters across all birds. J, Parameter error and SRMR as a function of sample size for simulations selected from 3 birds. Here, parameter error was taken as the median MAD across simulations and birds. Solid lines represent a regression of error and SRMR with the square root of sample size. K-N, Distribution of Pearson’s correlation between the real and simulated latent variables. O, Median latent variable correlation from the same 3 birds taken as a function of sample size. Color legend is the same as in panel J.

These Monte Carlo simulations allowed us to evaluate the reliability of the model in two distinct ways. First, we compared the overall fit of the timing variability model to the real vs. artificially generated data by comparing the model covariance calculated from equation (3) to the data covariance matrix for both the artificial and real data sets. In all birds, SRMR was smaller for the fit to the simulated data than to the actual data (Fig. 6E). Median SRMR computed across simulations ranged from 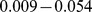 by bird, roughly two thirds of what we found in the real data. This suggests that about two thirds of the difference between real and model covariance matrices resulted from idiosyncratic components of the covariance matrix due to random sampling, while a third of the difference resulted from patterns in the data that did not conform to the assumptions of the model. It is unclear whether these patterns represent additional global timing factors, specific dependencies between the factors that are not included in the model, or limitations due to other model assumptions.

Second, we compared the parameters and latent variables from the best-fit model to the (known) parameters and latent variables in the artificial data set. This allowed us to assess the reliability of model estimates due purely to statistical variation in the timing of interval resulting from the posited latent factors. We report reliability using the median absolute deviation of estimates from the true values. In the 5 birds with multiple global parameters, we generated artificial data for both the single-global and multiple-global factor models; however, because error was similar across both approaches (see below) we focus on the multiple global factor models in order to include estimates of the 2nd global factor.

All parameters had similar magnitudes of error (Fig. 6F–I): across bird simulations, tempo parameter error was 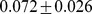 msec, while error in the 2nd global parameter was a 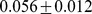 msec; independent parameter error, 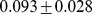 msec; and jitter error, 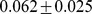 msec. In the five birds with multiple global factors, the average error was similar in the single-global factor model, with no significant differences among independent and jitter parameters (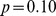 and 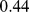 respectively, Wilcoxon signed-rank test), and a small albeit significant decrease of 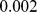 msec in error in the tempo-based parameters (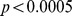, Wilcoxon signed-rank test). We also verified that the error in parameter estimates reliably decreased with the square root of sample size: For 3 representative birds we ran 200 simulations at each of several sample sizes ranging from 50 to 5000 (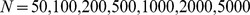). A regression of median parameter error with the square-root of sample size showed a qualitatively close fit (Fig. 6J). We also computed the dependency of SRMR on sample size and found a similar relationship. Overall, deviation between estimated and known parameters tended to fall below 10 percent as song sample size increased above 200–500.

We then examined the accuracy of trial-by-trial maximum likelihood estimates of latent variables by computing Pearson’s correlation between the real and simulated variables and taking the median across simulations (Fig. 6K–N). Global tempo showed remarkably strong correlations between 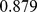 and 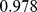 by bird (median 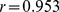), while the 2nd global factor showed a weaker correlation between 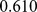 and 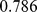 (median 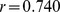). Across all time intervals, independent and jitter latent variables showed correlations of 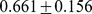 and 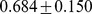. Interestingly, correlations were only weakly dependent on sample size and appeared to reach a ceiling by 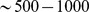 samples (Fig. 6O).

Finally, we tested the sensitivity of the model to the assumption that the underlying distributions of latent variables were Gaussian. To do this we reran simulations the same way as above (using actual sample sizes), except that for each simulation, and each latent variable, we randomly chose among a set of distributions that included a Gaussian and two Weibull distributions [30], one with significant negative skew (alpha = 10, beta = 2), and the other with positive skew (alpha = 2, beta = 10). Weibull distributions were mean-subtracted and scaled to have the same variance specified by the model. Interestingly, model fits and parameter error were similar to error when all distributions were Gaussian. SRMR ranged from 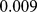 to 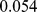 (median 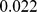). Tempo parameter error was 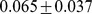 msec, while error in the 2nd global parameter was a 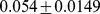 msec; independent parameter error, 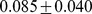 msec; and jitter error, 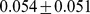 msec. Correlations with the latent variables were also similar, with a median global tempo correlation of 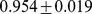, while the 2nd global correlation median was 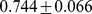, independent, 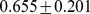 and jitter 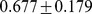. We next tested a more extreme violation of the Gaussian assumption by assigning exponential distributions to all latent variables (mean-subtracted and scaled as above). Even in this case model error was similar to when all distributions were Gaussian, with SRMR between 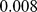 and 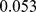 (median 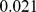); respective errors among the tempo, second global, independent and jitter parameters of 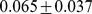, 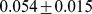, 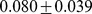 and 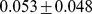 msec; and respective correlations with latent variables of 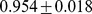, 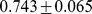, 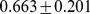 and 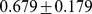.

Thus, the simulations indicated that the model was reasonably accurate at estimating parameters and trial-to-trial values of the latent variables, and robust to violations of the Gaussian assumption.

## Discussion

We have presented a statistical model of action sequences that separates trial-to-trial variability in global timing, uncorrelated variation in the duration of individual sequence elements, and timing jitter at the boundaries between elements. We derived an algorithm based on expectation-maximization to find maximum likelihood estimates of both model parameters and trial-to-trial values of each latent factor. Applying the model to the songs of 11 adult male zebra finches, we found that the model provided very good fits to trial-to-trial variability in the durations of song syllables and the gaps of silence between them, with each of the three factors contributing roughly equal amounts of variability to individual song elements. In contrast, measurements of motif length were dominated by the global factor since such variability accumulates in a correlated manner across song elements. Given that the neural mechanisms driving global variability are likely to be distinct from those driving both independent and jitter factors, experiments designed to evaluate the effect of behavioral or pharmacological manipulations of the system on timing variability may reach different conclusion based on whether timing variability is evaluated at the level of individual syllables or entire motifs [8].

Our timing variability model can be seen as an extension of the Wing-Kristofferson [16] model of timing in humans. The latter model has been used to investigate motor variability in studies where human subjects produced isochronous timing intervals via finger taps or other basic gestures [16], [25]–[28], [31], [32]. The key insight of Wing and Kristofferson [16] was to exploit the fact that variability in event timing that is introduced downstream of the motor pattern generator necessarily introduces negative covariance between the intervals surrounding the event. This insight was used to separate the measured timing variance into a component that was attributed to the central clock, and motor variability introduced at the motor periphery. In general, clock variability scales with the duration of the interval, consistent with a process of accumulating variability of the time course of the interval, while motor variability shows little or no change with duration, consistent with variability introduced in a specific event that does not accumulate over time [16]. Consistent with these ideas, we were able to use negative covariance to segregate out a component of timing variance in zebra finch song that did not scale with interval duration. In contrast, the global and independent timing variability, hypothesized to relate to central circuits for song generation, did scale. Importantly, previous extensions of the Wing-Kristofferson model have also included a “tempo” variable, in which the speed of tapping slowly drifts over the course of an experiment [31]–[33].

In contrast to the original Wing-Kristofferson model and its extensions, the timing variability model yields data on heterogenous action sequences and can measure how variability in each latent factor is spread across different sequence elements. Indeed, we found prominent and systematic differences in the variability of syllables and inter-syllable gaps. We also found that the gaps between motifs were more variable than within-motif gaps, and that this enhanced variability is expressed only in the independent component of timing variability, not in its sensitivity to global timing. Given the millisecond scale linkage between the timing of neural activity and song behavior [9]–[11], these data may be used to constrain mechanistic models of song production.

These findings may bear on how precise patterns of neural activity remain synchronized over disparate song nuclei. While several studies have suggested that HVC controls the timing of all neural activity [11], [13], [34], [35], both left and right HVC appear to make independent contributions to song tempo [13]. Moreover, lesions to LMAN, the output of a basal ganglia circuit, decrease variability in syllable duration [8], suggesting that this nucleus contributes to timing variability as well. One idea for how areas remain in temporal alignment comes from electrophysiological evidence that left and right HVC are synchronized at specific time points within the song, and generally near syllable onsets [36]. Thus, while each HVC could run at a slightly different tempo, they may be periodically reset to a global tempo. If such resetting occurs preferentially at syllable onsets, this in turn would lead to a tradeoff between the durations of syllables and gaps, with the magnitude of the tradeoff depending on the mismatch between HVC and global tempo. If this mismatch were to undergo slow changes over time, then the tradeoff between syllable and gap lengths would be quite similar to the properties of the second global factor in our behavioral data: it would not scale with syllable length but would show circadian modulation and slow drift. It may be the case that the tempo mismatch is smaller in some birds than others, explaining why the second factor reached significance in just 5 of 11 birds. This notion of a reset is consistent with recent recordings of air sac pressure during gaps, in which ongoing patterns of negative pressure are rapidly terminated near syllable onsets [12]. Finally, there is electrophysiological evidence for a separate source of signals that triggers the onset of motifs [29], which would be consistent with the especially large independent timing variability we find in inter-motif gaps because that source could introduce additional variability to the onset time of the first motif syllable.

In using the EM algorithm to separate the three hypothesized components of timing variance, the timing variability model also yields maximum likelihood estimates of the latent variables on a trial-to-trial basis. These values can then be related to variables that influence song timing but were not included in the original model. Here we have used this approach to quantify slow drift in timing that occurs on timescales much longer than between the production of individual songs. It is important to note that the original data used for this model consisted of songs collected in a variety of behavioral conditions (males were either alone, or singing in the presence of another adult or juvenile male). Both drift, as well as the magnitude of variability components, could conceivably be affected by this factor; future investigations could either track latent variables across conditions or estimate parameters separately for each. Regardless, timing drift has been previously been reported in zebra finch song, and was shown to be highly correlated with drift in the duration of interburst intervals in the song nucleus RA [10]. That study did not decompose variability into different sources; it would be interesting to examine whether the tight correlation specifically stems from any of the individual components we have presented here.

Our use of EM for timing analysis falls within a broader theoretical effort in fitting probabilistic generative models to data [17], [37]. This work suggests several extensions to the current timing variability model, including the use of Gaussian process or Kalman filters to explicitly model slower trial-by-trial drift in timing [37], [38]. Another advantage of the generative model approach is that Monte Carlo simulations can be used to understand the reliability and limitations of the underlying model. Here we have examined the statistical reliability of the model by generating artificial data sets and then fitting the model to these data. Further insight into the robustness of the model can be obtained by altering key model assumptions, generating artificial data sets with the altered model, and then examining how well the original model fits these altered data. We have used this approach to argue that the model is robust to alterations in the assumption that the shape of the latent variable distribution is Gaussian. Further such investigations are beyond the scope of the present paper.

Behavioral and neural variability are often affected by multiple sources whose effects are shared by many different areas of the given system. Generative models address this problem by assigning latent variables to these different sources, and thus have the potential to help quantify the activity of hypothesized synergies in sensorimotor systems [39]–[41]. For precisely timed tasks such as zebra finch song, the coordination of activity throughout the system is a challenge, and the examination of timing variability may be particularly valuable in understanding the functional organization of the song circuit. By applying the methods developed here to a joint analysis of neural and behavioral timing, future studies may yield a more complete understanding of the mechanisms linking neural activity and song behavior.

## Methods

### Ethics Statement

All bird care and housing was approved by the institutional animal care and use committee at the University of Maryland, College Park.

### Model and Derivation

We fit the timing variability model using an EM algorithm similar to what has been used for for maximum likelihood factor analysis [17], 23,42. We begin by rewriting equations 2 and 3 for the row-vector of mean-subtracted interval durations for sample 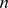, and the resulting model covariance matrix 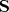:
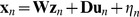
(4)

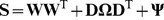
(5)where 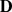 is the differencing matrix described in Results. We will use 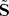 to denote the data covariance matrix, in order to distinguish it from 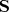.

The free parameters of the model are the global weights 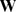 and the expected jitter and independent variances along the diagonals of 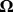 and 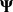. We use vector 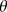 to refer to the entire set of these parameters. For 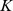 time intervals in a sequence, 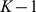 modeled boundaries between intervals, and 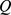 global variables, the dimensions of 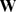 are 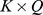; 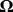, 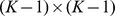; and 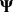, 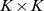. The total number of parameters in the model is thus 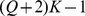. In the original formulation of the model we assumed a single global dimension representing tempo, so in that case 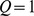 and the total number of parameters is 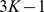.

For a given parameter set, the model can be run in generative mode in which latent variables 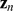, 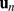, and 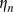 are generated and then combined to according to equation (4) to produce mean-subtracted interval durations 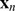. Our goal is to find a set of parameters 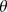 that maximizes the likelihood of generating the set of interval durations that were actually observed, *i.e.* we want to maximize 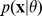. Although there is no closed-form solution to this problem in general, EM provides an iterative procedure for changing parameters so that each step of the algorithm increases the probability of generating the real data, *i.e.* 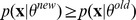.

Our derivation considers the independent variables 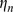 separately from the global and jitter variables 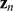 and 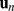, and concatenating the latter two into a single 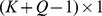 row vector 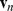. We also let 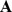 denote the 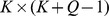 matrix obtained by concatenating 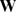 and 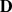 and let 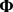 denote the 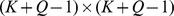 expected covariance matrix for 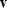:
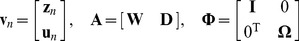
(6)where 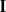 is the 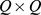 identity matrix, representing the assumed unit covariance of 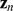, and 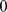 is a 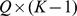 matrix of zeroes. So 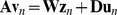 and


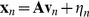
(7)


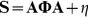
(8)

Equation 8 may be viewed as a kind of “confirmatory factor analysis” or “covariance structure analysis” [18], [19], [21], [22], which are generalizations of factor analysis in which 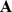 and 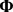 contain a combination of free and fixed parameters.

The basic idea in the EM algorithm stems from obtaining the probability we seek to maximize, 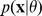, by marginalizing over the latent variables 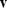, *i.e.* 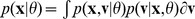. Instead of dealing with both terms in this integral at the same time, EM is an iterative algorithm that addresses each term separately. In the ‘E’ step, one fixes the parameters and finds the probability distribution (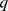) of latent variables in each sample at the current values of the parameters, 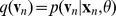. The structure of the model implies that the conditional distribution of the data 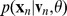 is a Gaussian with mean 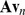 and covariance matrix equal to the independent covariance matrix 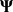. From Bayes’ Theorem for Gaussian variables [17], it follows that 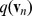 is also a Gaussian with covariance defined by
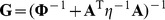
(9)and expected mean
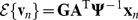
(10)where we use the notation 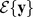 to denote the expected value of any given variable 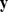 under the distribution 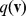. We also require the 2nd moment of 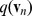, which is given by
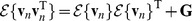
(11)using the basic result that 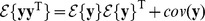.

In order to analyze the global and jitter variables separately we can simply separate out the expected values from 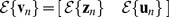. Below we will also make use of the 2nd moments of the original latent variables and use the fact that
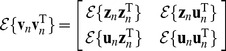
(12)


It is important to note that while the latent variables are independent of each other by definition, they are not necessarily independent under the posterior distribution 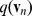. For example, 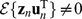. Thus, while we seek to separate the global and jitter parameters as well as corresponding latent variables, the E step is facilitated by their concatenation.

In the ‘M’ step, one finds new values for 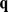 that maximize the expected log likelihood of the joint distribution of the data and the latent variables under 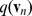. That is, we want to find 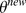 that maximizes
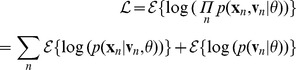
(13)


Using the standard log likelihood function of a multivariate Gaussian distribution, along with our definition of 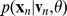 as a Gaussian with mean 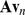 and covariance matrix 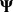,
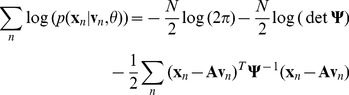
(14)


Conditioned solely on the parameters 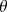, the distribution of 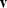 is also Gaussian with covariance matrix 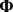. So
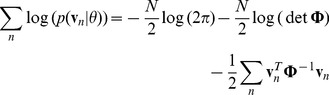
(15)


We seek values of 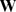, 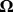, and 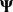 that maximize 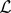. Thus, for each variable, we take the partial derivative of 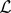 with respect to the variable, set the derivative equal to zero and solve for the variable. Below we will make use of the fact that taking expected values is a linear operation, so the derivative of the expected value is equal to the expected value of the derivative.

We begin with 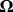, noting that the variable is contained in 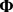 so the partial derivative only depends on the last two terms of equation 15. Since 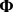 is simply a concatenation that includes diagonal matrices 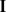 and 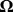 as in equation 6, we can make use of the fact that 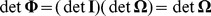. Also, 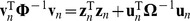, and we can discard the term with 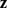 for the partial derivative. Therefore,
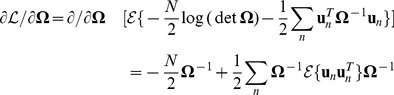
(16)which makes use of the identity 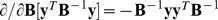 when 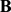 is symmetric. Setting the derivative to zero and solving for 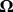 we have



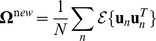
(17)This is simply the standard solution for the expected 2nd moment of any Gaussian distribution (in this case 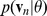) and follows directly from the fact that the partial derivative does not depend on any terms in 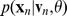.

To maximize 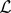 with respect to 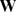 we first note that the variable is contained in 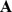, so the only term that is relevant to the partial derivative is the quadratic in equation 14, which can be rewritten as 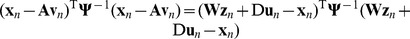. We also make use of the identity 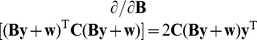, to give
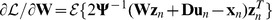
(18)


Setting the derivative to 0 and solving for 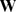, we have
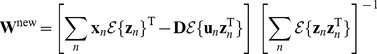
(19)


To maximize 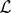 with respect to 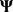 we require the first two terms of equation 15 and use 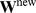 as well as the identity used to derive 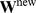, finding:
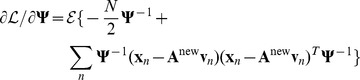
(20)where 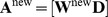.

Setting the expected value of the partial derivative to zero and solving for 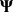:
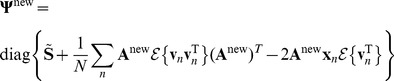
(21)where we have used 
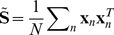
 and “diag” is the operation that creates a diagonal matrix by setting all off-diagonal elements to zero.

### Model Implementation

We implemented the model with custom code written for Matlab (Mathworks, Natick, MA). Like all “hill-climbing” algorithms, EM is subject to getting stuck in a local maximum. Therefore, for each optimization we ran EM 100 times starting from different sets of initial parameter values, chosen from a uniform distribution ranging from zero to a maximum value determined by the variability of the corresponding interval. For the global weight 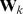 initial values ranged from 0 to the standard deviation of 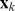. Initial values of the independent variance for interval 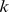 ranged from 0 to the sample variance of that interval. Since a jitter parameters are not associated with unique intervals and overall contribute twice as much variance to intervals as other parameters, initial values for the jitter variances ranged from 0 to half of average interval variance. For each set of initial conditions, we stopped the algorithm when it satisfied the relatively loose convergence criterion that the log-likelihood of the data given the parameters (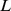) increase by 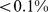 over a given iteration. Here, 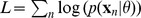, which is a Gaussian with a mean of 0 and covariance 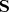. Using the standard formula for the likelihood function of a Gaussian and the fact that 
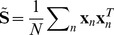
, it is easily verified that
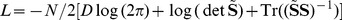
(22)


After running the algorithm from 100 initial conditions we picked the 5 parameter sets with the highest log-likelihoods and continued running the algorithm on those parameters until the global and timing jitter parameters changed by 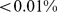 from the previous iteration. We omitted independent variability because we expected that parameter set to converge more quickly; we have verified that assumption and note that there is no significant difference in parameter estimates or error in Monte Carlo simulations when independent parameters are directly required to converge in the code. After parameters converged, we then picked the parameter set associated with the highest log-likelihood under that criterion. Across song data and Monte Carlo simulations the algorithm never failed to converge within a 10,000 iteration limit specified in the computer code. The number of iterations required to meet the looser convergence criterion typically ranged from 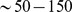, while for the stricter criterion the range was 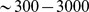 with the large majority being 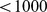.

#### Model fit evaluation

Model fits were evaluated using the standardized Root Mean Squared Residual (SRMR) between the data and model correlation matrices [24]. We let the residual correlation 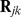 for time intervals 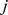 and *k* equal the difference between Pearson’s correlation coefficient for *j* and *k* determined from the data, minus the covariance of these intervals predicted by the modeled normalized by the standard deviations in the data (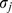 and 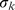):
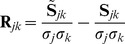
(23)


SRMR is just the root mean square average of 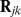, where we only consider values with 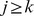 since 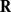 is symmetric:
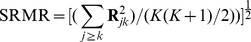
(24)


#### Additional global factors

Initially, all model fits were performed with only 1 global timing factor, 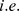 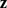 is one dimensional. However, the song data yielded a few SRMR values that we considered relatively large, and a qualitative examination suggested that significant structure in the off-diagonal elements of the data covariance matrix was not being captured by the model. We thus considered additional global timing factors by increasing the number of dimensions (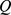) in 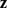. Here, each dimension of 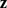 remains unit-Gaussian and is independent of the other dimensions, so the expected covariance of 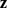 is the 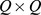 identity matrix. The derivation of the EM algorithm is unchanged; 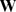 is simply a 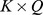 dimensional matrix rather than a 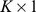 dimensional column vector.

To determine the optimal number of global factors per bird, we ran the entire EM algorithm (including 100 initial conditions) for each value of 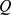 ranging from 1–4. We then computed the Bayesian Information Criterion (BIC) [43], and chose the dimensionality associated with the lowest BIC. Specifically, we calculated BIC as 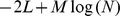 where 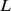 is the log-likelihood defined in equation 22, 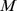 is the total number of model parameters 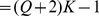 and 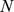 is the number of samples as before.

For a number of birds the BIC analysis indicated that 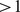 global timing factor provided the best fit to the data. However, the columns of 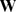, and hence the global factor weights are not uniquely specified by the algorithm: multiple estimates of 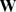 can yield the same global covariance matrix 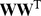. This interpretive difficulty is common in factor analysis and is typically addressed using various transformations, called factor rotations, that are based on a priori assumptions about the relationship among latent variables. Here, we consider an orthonormal transformation (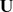) in latent variable space. In this new basis, the transformation from latent to observable space is given by the matrix 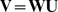, so 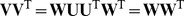.

We chose a transformation 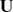 such that all the global variability in total sequence length was captured by the first dimension in latent variable space. For any latent vector 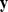, the global contribution to total sequence length can be determined by taking the inner product of the image of 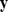 under 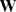 with the 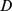 dimensional column vector of ones 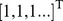, which we designate as 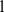, *i.e.* global sequence length is 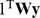. Thus, all global variation in sequence length is determined by variation in the direction of the unit-length latent vector
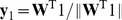
(25)


Importantly, any other direction 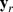 in latent variable space that is orthogonal to 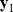 will yield no variation in global sequence length: by definition of 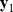, this is equal to 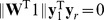. Therefore, a rotation matrix 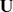 whose first column is given by 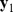 yields a transformation where all global sequence length variation is confined to the first factor, and the first column of 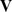 should be the image of 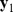 under the transformation 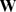: 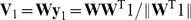.

For 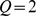 global factors, we need only to complete the basis by setting the second column of 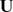 to be a unit vector in the unique direction perpendicular to 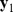. However, for the one bird with 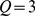, there were an infinite number of solutions since there is no unique orthonormal basis to the remaining 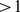 dimensional latent space, so we sought a more general solution. Thus, to fill out the remaining columns of 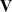, we subtracted off the variations in the direction of 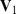 by calculating the covariance matrix 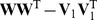. We then performed a principal components analysis on this covariance matrix by computing 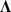, the 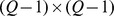 diagonal matrix of non-zero eigenvalues, and 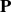, the 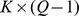 matrix of associated eigenvectors. Then we set
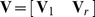
(26)


where
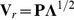
(27)


By convention we can sort the columns of 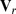 from largest to smallest eigenvalue. Thus, when comparing the 2nd columns of 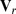 across birds we are effectively choosing the dimensions that maximize the sum of the global data variances that remain after subtracting out global sequence length variability. Note that by construction, 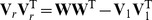, ensuring that 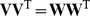.

### Song Data

We used song data from 11 adult zebra finch males >400 days-post hatch. All care and housing was approved by the institutional animal care and use committee at the University of Maryland, College Park. Our basic procedure for collecting and extracting song data was based on custom code written in Matlab and has been previously reported in [14], [15]; we provide a brief description below.

Recordings were made from sound isolation chambers (Industrial Acoustics, Bronx, NY), which contained two cages separated by 18 cm and two directional microphones (Pro 45; Audio-Technica, Stow, Ohio). Signals were digitized at 24,414.1 Hz, and ongoing data were selected using a circular buffer and a sliding window amplitude algorithm. “Sound clips” separated by <200 msec were included in the same “recording” and clip onset times were indicated by filling the gaps between clips with zeros.

For each bird, we gathered an initial random sample of 1000 recordings >2 sec long and had maximum power from the side on which the target bird was housed. We analyzed recordings using the log-amplitude of the fast-Fourier transform (FFT) with a 256-point (10.49 msec) window moved forward in 128-point steps and excluded frequencies outside the 1.7–9 kHz range from all subsequent analysis because song structure is less reliable outside that range. We then used an automated template-matching algorithm [14] to identify individual song syllables, and selected out recordings which contained repeated sequences of the most commonly produced motif. Our final sample of songs ranged from 215–885 per bird.

After we identified syllables and syllable sequences we measured the onset and offset times of each syllable with a more fine-grained algorithm: First, we recalculated spectrograms from the original signal using FFTs with a 128-point window slid forward in 4-point steps, yielding 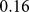 msec time bins. Although previous research [14], [15] had been based on log-amplitudes, for this study we used raw amplitudes, which we have found to be more reliable for timing measurements. We then computed time-derivative spectrograms as differences in amplitude in time-adjacent bins. We next smoothed in time the resulting spectrograms with a 64-point Gaussian window that had a 25.6-point (∼5 msec) standard deviation. This windowing was found to be a good compromise between the competing demands of averaging out extraneous peaks and troughs in the time-derivative vs. preserving the peaks and troughs that we had defined as syllable onsets and offsets.

Finally, for each syllable we constructed a new template based on the time-derivative spectrogram and used a novel dynamic time-warping algorithm to determine the precise times at which onsets and offsets occurred in individual recordings of those syllables; see [14] especially for more detail on this process. Onsets/offsets were generally identified as salient time-derivative peaks/troughs in the template spectrogram; these correspond to inflection points in the rise and fall of energy at the beginning and ends of syllables.

## Supporting Information

Matlab S1 A.zip file containing Matlab functions for model implementation. Also contains a.txt file giving an overview of the functions.
(ZIP)
