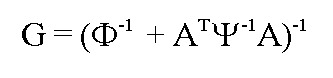# Correction: A Generative Model for Measuring Latent Timing Structure in Motor Sequences

**DOI:** 10.1371/annotation/ae2a9c7b-37fd-4aae-a9df-edbf8e7d99b8

**Published:** 2012-10-25

**Authors:** Christopher M. Glaze, Todd W. Troyer

There was a formatting error in Equation 9. The correct Equation 9 can be viewed here: